# Adipocyte calcium sensing receptor is not involved in visceral adipose tissue inflammation or atherosclerosis development in hyperlipidemic *Apoe*^*−/−*^ mice

**DOI:** 10.1038/s41598-021-89893-y

**Published:** 2021-05-17

**Authors:** Sai Sahana Sundararaman, Linsey J. F. Peters, Yvonne Jansen, Selin Gencer, Yi Yan, Sumra Nazir, Andrea Bonnin Marquez, Florian Kahles, Michael Lehrke, Erik A. L. Biessen, Joachim Jankowski, Christian Weber, Yvonne Döring, Emiel P. C. van der Vorst

**Affiliations:** 1 Interdisciplinary Center for Clinical Research (IZKF), RWTH Aachen University, Pauwelsstrasse 30, 52074 Aachen, Germany; 2 Institute for Molecular Cardiovascular Research (IMCAR), RWTH Aachen University, Aachen, Germany; 3 Department of Pathology, Cardiovascular Research Institute Maastricht (CARIM), Maastricht University Medical Centre, Maastricht, The Netherlands; 4 Institute for Cardiovascular Prevention (IPEK), Ludwig-Maximilians-University Munich, Munich, Germany; 5 DZHK (German Centre for Cardiovascular Research), Partner Site Munich Heart Alliance, Munich, Germany; 6 Department of Internal Medicine I-Cardiology, University Hospital Aachen, Aachen, Germany; 7 Department of Biochemistry, Cardiovascular Research Institute Maastricht (CARIM), Maastricht University Medical Centre, Maastricht, The Netherlands; 8 Munich Cluster for Systems Neurology (SyNergy), Munich, Germany; 9 Department of Angiology, Swiss Cardiovascular Center, Inselspital, Bern University Hospital, University of Bern, Bern, Switzerland

**Keywords:** Cardiovascular biology, Mechanisms of disease

## Abstract

The calcium sensing receptor (CaSR) is a G-protein coupled receptor that especially plays an important role in the sensing of extracellular calcium to maintain its homeostasis. Several in-vitro studies demonstrated that CaSR plays a role in adipose tissue metabolism and inflammation, resulting in systemic inflammation and contributing to atherosclerosis development. The aim of this study was to investigate whether adipocyte CaSR plays a role in adipose tissue inflammation in-vivo and atherosclerosis development. By using a newly established conditional mature adipocyte specific CaSR deficient mouse on a hyperlipidemic and atherosclerosis prone *Apoe*^*−/−*^ background it could be shown that CaSR deficiency in adipocytes does neither contribute to initiation nor to progression of atherosclerotic plaques as judged by the unchanged lesion size or composition. Additionally, CaSR deficiency did not influence gonadal visceral adipose tissue (vAT) inflammation in-vivo, although a small decrease in gonadal visceral adipose cholesterol content could be observed. In conclusion, adipocyte CaSR seems not to be involved in vAT inflammation in-vivo and does not influence atherosclerosis development in hyperlipidemic Apoe^−/−^ mice.

## Introduction

Atherosclerosis, a progressive chronic inflammatory disease, is the most common cause of cardiovascular diseases^1^. High concentrations of lipids (hyperlipidemia), especially low density lipoprotein (LDL) are one of the main risk factors and contributors to disease progression^2^. Upon stimulation, adipose tissues enlarge and release many different products, like adipokines^3,4^, chemokines^5,6^, fatty acids, micro-particles and reactive oxygen species that have a wide range of targets. One of these targets is the cardiovascular system leading to vascular inflammation, accumulation of lipids and atherosclerosis^7^. The adipose tissue secretome is tightly controlled by complex homeostatic control mechanisms^8^. Adipose tissue can become dysfunctional in pathologies like obesity and hypercholesterolemia^8^.

In line with this, the activation of the calcium-sensing receptor (CaSR) elevates, at least in-vitro, the expression of pro-inflammatory factors in human adipocytes and adipose tissue in a NF-κB dependent manner^9^. A self-reinforcing feedback loop exists in which expression of *Casr* increases due to pro-inflammatory cytokines^10^. CaSR was discovered a quarter century ago^11^ and has been shown to play a crucial role in calcium homeostasis in the human body^12,13^. Not surprisingly, CaSR is therefore also expressed on the cell surfaces of various important organs in the calcium metabolism like parathyroid gland^14,15^, bone, kidney^16^, gut^17,18^ and skin^19^. The receptor is a G-protein coupled receptor that is made of 1078 amino acid residues and has 3 structural domains of which the largest domain recognizes calcium ions^20,21^. The receptor, especially in the parathyroid gland, senses even the smallest changes in circulating calcium ions and uses feedback loops to maintain calcium homeostasis^22^. Besides maintaining calcium homeostasis, CaSR obviously also plays a role in various other processes, like inflammation^23^. Furthermore, it was shown that CaSR activation promotes pre-adipocyte differentiation, adipogenesis and adipocyte differentiation^24^, while inhibiting lipolysis^25^. Thus, it is conceivable that CaSR plays a role in both adipose tissue inflammation and metabolism, although this remains to be validated in an in-vivo setting.

As adipose tissue builds-up and inflammation contributes to systemic inflammation^8^, it is likely that stimulation of these processes by CaSR plays a role in atherosclerosis development. Therefore, we generated mature adipocyte specific CaSR deficient mice on an atherosclerosis prone background, to determine whether adipocyte CaSR indeed exacerbates atherosclerosis development by stimulating adipose tissue inflammation in-vivo.

## Results

### Adipocyte specific CaSR deficiency does not affect early plaque size or phenotype

**Figure 1:**
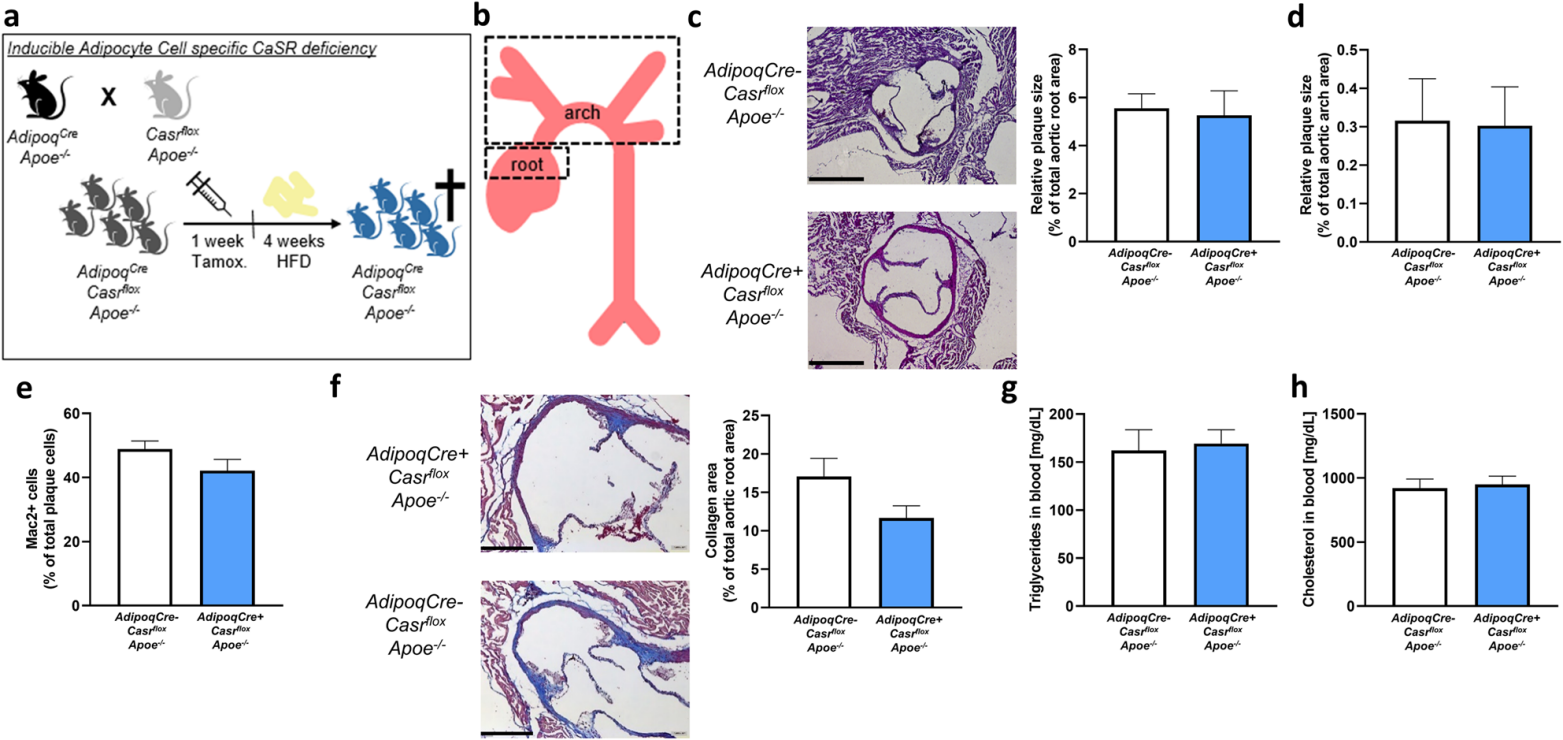
Adipocyte specific CaSR deficiency does not influence early atherosclerotic lesion development. (**a**) Representation of the experimental workflow. Mice were crossed and offspring were injected with tamoxifen and fed a HFD for 4 weeks before analysis. (**b**) Schematic representation of the areas that were used for analysing atherosclerotic plaques. (**c**) Representative images and quantification of aortic root lesions in both *AdipoqCre− Casr*^*flox*^* Apoe*^*−/−*^ and *AdipoqCre*+ *Casr*^*flox*^* Apoe*^*−/−*^ mice (n = 10–12). Scale bar 500 µm. (**d**) Quantification of aortic arch lesions in *AdipoqCre− Casr*^*flox*^* Apoe*^*−/−*^ and *AdipoqCre*+ *Casr*^*flox*^* Apoe*^*−/−*^ mice (n = 10–15). (**e**) Quantification of macrophage content of aortic root lesions in *AdipoqCre− Casr*^*flox*^* Apoe*^*−/−*^ and *AdipoqCre*+ *Casr*^*flox*^* Apoe*^*−/−*^ mice (n = 11–13). (**f**) Images to represent the presence of collagen in aortic root lesions and quantification in *AdipoqCre− Casr*^*flox*^* Apoe*^*−/−*^ and *AdipoqCre*+ *Casr*^*flox*^* Apoe*^*−/−*^ mice (n = 11–12). Scale bar 250 µm. (**g**,**h**) Quantification of plasma triglycerides (**g**) and cholesterol (**h**) levels of *AdipoqCre− Casr*^*flox*^* Apoe*^*−/−*^ and *AdipoqCre*+ *Casr*^*flox*^* Apoe*^*−/−*^ mice (n = 10–15). Image processing was done using Image J 1.53 (https://imagej.nih.gov/ij/). Bar graphs are representation of mean ± SEM.

To investigate the role of adipocyte CaSR on atherogenesis, *AdipoqCre*+ *Casr*^*flox*^* Apoe*^*−/−*^ and *AdipoqCre− Casr*^*flox*^* Apoe*^*−/−*^ (control) mice were injected with tamoxifen (to induce Cre expression in mature adipocytes) and fed a high-fat diet (HFD) for 4 weeks (Fig. 1a). Subsequently, atherosclerotic lesion sizes were analysed in the aortic roots and aortic arches (Fig. 1b). No difference was observed in the lesion sizes between *AdipoqCre*+ *Casr*^*flox*^* Apoe*^*−/−*^ and *AdipoqCre− Casr*^*flox*^* Apoe*^*−/−*^ mice, neither in aortic roots nor arches (Fig. 1c,d). Analyses of the lesion composition demonstrated that the relative macrophage content in the plaque did not change in adipocyte CaSR deficient mice, compared to controls (Fig. 1e). Furthermore, collagen content was not changed upon adipocyte CaSR deficiency (Fig. 1f). Systemically, adipocyte CaSR deficiency did not change total plasma triglycerides or cholesterol levels (Fig. 1g,h). Flow cytometry analysis of the blood also revealed no changes in circulating leukocyte numbers and profile, platelet counts or body weight (Table 1).

**Table 1:** Body weight and immune cells in blood in mice fed with HFD for 4 weeks.

4 weeks HFD	*AdipoqCre−* *Casr*^*flox*^ *Apoe*^*−/−*^ (n = 13–15)	*AdipoqCre*+ *Casr*^*flox*^ *Apoe*^*−/−*^ (n = 9–10)	p value
Leukocytes [× 10^6^/ml]	1.9 ± 0.2	2.4 ± 0.5	0.3789
Neutrophils [× 10^5^/ml]	6.6 ± 0.8	5.6 ± 1.0	0.6850
Classical monocytes [× 10^5^/ml]	1.7 ± 0.2	1.8 ± 0.6	0.9763
Non-classical monocytes [× 10^4^/ml]	8.1 ± 1.3	5.6 ± 1.0	0.3268
B cells [× 10^5^/ml]	5.7 ± 0.7	5.2 ± 0.7	0.8776
T cells [× 10^4^/ml]	2.2 ± 0.3	2.4 ± 0.3	0.9327
Thrombocytes [× 10^3^/µl]	588 ± 70	839 ± 92	0.8719
Body weight (g)	26.8 ± 1.0	27.2 ± 1.4	0.8159

Analysis has been done using FACS Diva software 8.0.3 (www.bdbiosciences.com) and FlowJo 10.7 (www.flowjo.com).

### CaSR deficiency in adipocytes does not affect advanced plaque size or phenotype

**Figure 2:**
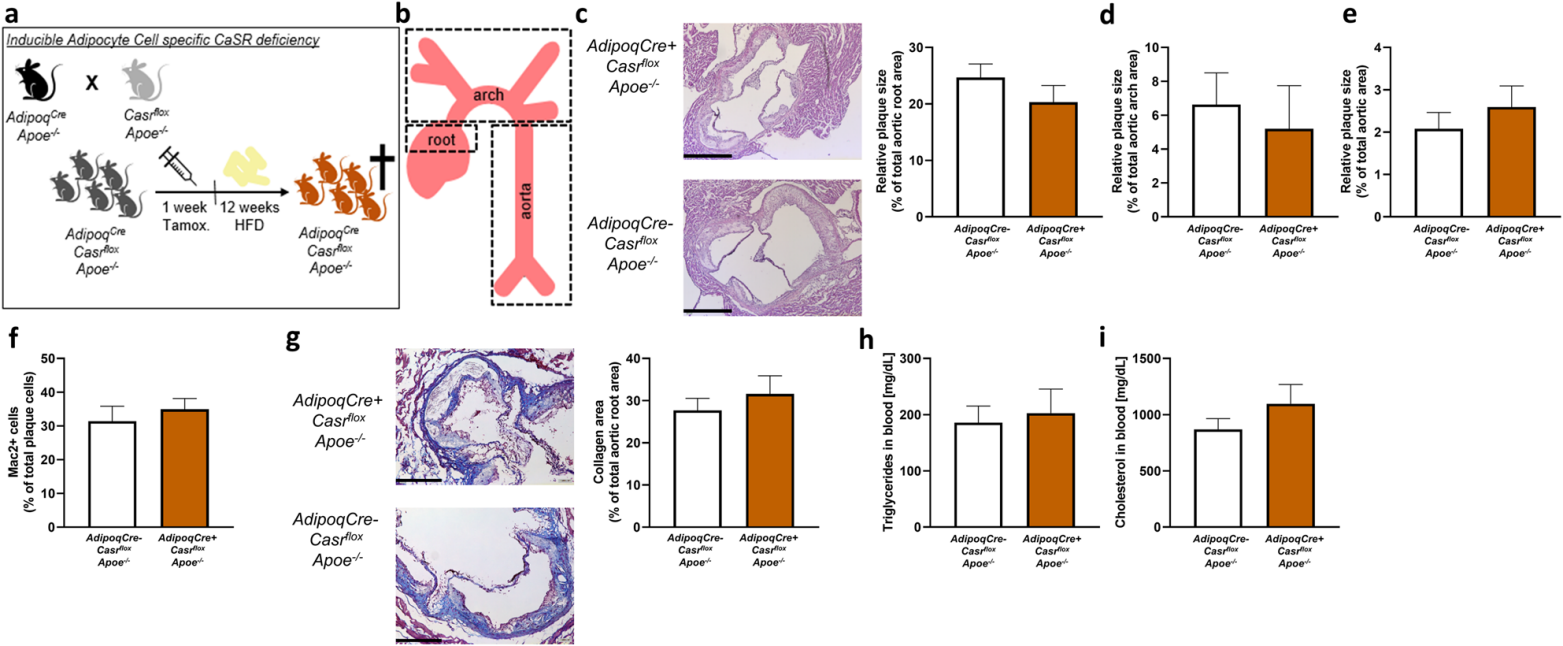
Adipocyte specific CaSR deficiency does not influence advanced stages of atherosclerosis. (**a**) Representation of the experimental workflow. Mice were crossed and offspring were injected with tamoxifen and fed a HFD for 12 weeks before analysis. (**b**) Schematic representation of the areas that were used for analysing atherosclerotic plaques. (**c**) Representative images and quantification of aortic root lesions in both *AdipoqCre− Casr*^*flox*^* Apoe*^*−/−*^ and *AdipoqCre*+ *Casr*^*flox*^* Apoe*^*−/−*^ mice (n = 9–10). Scale bar 500 µm. (**d**,**e**) Quantification of aortic arch lesions (n = 5) (**d**) and thoraco-abdominal aortic lesions (n = 9–10) (**e**) in *AdipoqCre− Casr*^*flox*^* Apoe*^*−/−*^ and *AdipoqCre*+ *Casr*^*flox*^* Apoe*^*−/−*^ mice. (**f**) Quantification of macrophage content of aortic root lesions in *AdipoqCre− Casr*^*flox*^* Apoe*^*−/−*^ and *AdipoqCre*+ *Casr*^*flox*^* Apoe*^*−/−*^ mice (n = 9). (**g**) Images to represent the presence of collagen in plaques in aortic roots and their quantification in *AdipoqCre− Casr*^*flox*^* Apoe*^*−/−*^ and *AdipoqCre*+ *Casr*^*flox*^* Apoe*^*−/−*^ mice (n = 10). Scale bar 250 µm. (**h**,**i**) Quantification of plasma triglycerides (**h**) and cholesterol (**i**) levels of *AdipoqCre− Casr*^*flox*^* Apoe*^*−/−*^ and *AdipoqCre*+ *Casr*^*flox*^* Apoe*^*−/−*^ mice (n = 10–11). Image processing was done using Image J 1.53 (https://imagej.nih.gov/ij/). Bar graphs are representation of mean ± SEM.

Although adipocyte CaSR did not influence atherogenesis, it is plausible that potential effects only become evident during later stages of disease development when adipose tissues enlarge and become dysfunctional. Therefore, adipocyte specific CaSR deficient mice and matching controls were fed for 12 weeks with a HFD to investigate the effects on advanced atherosclerotic lesion formation (Fig. 2a). Subsequently, aortic lesions were analysed in the aortic roots, aortic arches and thoraco-abdominal aorta (Fig. 2b). No differences could be observed in the lesion sizes between *AdipoqCre*+ *Casr*^*flox*^* Apoe*^*−/−*^ and *AdipoqCre− Casr*^*flox*^* Apoe*^*−/−*^ mice in either of the investigated vascular locations (Fig. 2c–e). Furthermore, lesion phenotyping in the aortic roots demonstrated that there was no difference in relative macrophage content or collagen content, comparing adipocyte specific CaSR deficient mice with controls (Fig. 2f,g). Plasma total cholesterol and triglycerides levels were also not changed upon adipocyte CaSR deficiency (Fig. 2h,i). There also was no difference in circulating leukocyte numbers and profile, platelet counts or body weight (Table 2).

**Table 2:** Body weight and immune cells in blood in mice fed with HFD for 12 weeks.

12 weeks HFD	*AdipoqCre−* *Casr*^*flox*^ *Apoe*^*−/−*^ (n = 11)	*AdipoqCre*+ *Casr*^*flox*^ *Apoe*^*−/−*^ (n = 9–10)	p value
Leukocytes [× 10^6^/ml]	1.6 ± 0.5	1.1 ± 0.4	0.5239
Neutrophils [× 10^5^/ml]	9.9 ± 2.6	6.9 ± 1.1	0.3100
Classical monocytes [× 10^5^/ml]	1.6 ± 0.2	1.2 ± 0.3	0.3920
Non-classical monocytes [× 10^4^/ml]	8.6 ± 1.5	10.3 ± 3.1	0.6269
B cells [× 10^5^/ml]	1.2 ± 0.2	1.1 ± 0.3	0.7920
T cells [× 10^4^/ml]	5.1 ± 0.9	4.7 ± 1.5	0.8346
Thrombocytes [× 10^3^/µl]	1120 ± 94	1085 ± 135	0.8316
Body weight (g)	21.86 ± 1.0	23.86 ± 1.0	0.264

Analysis has been done using FACS Diva software 8.0.3 (www.bdbiosciences.com) and FlowJo 10.7 (www.flowjo.com).

### Deficiency of CaSR in adipocytes does not affect vAT inflammation in-vivo

**Figure 3:**
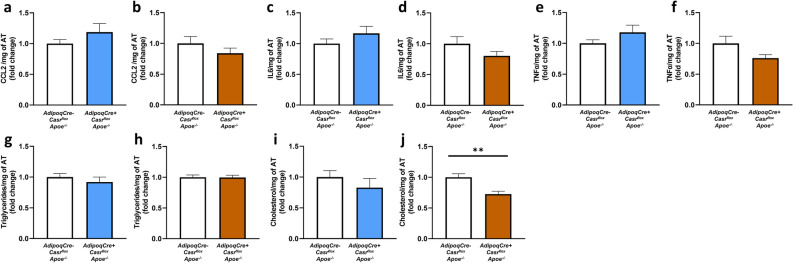
CaSR in adipocytes does not interfere with vAT inflammation in-vivo. (**a**–**f**) Inflammatory cytokines CCL2 (**a**,**b**), IL6 (**c**,**d**) and TNF-α (**e**,**f**) were measured in vAT isolated from *AdipoqCre− Casr*^*flox*^* Apoe*^*−/−*^ and *AdipoqCre*+ *Casr*^*flox*^* Apoe*^*−/−*^ after 4 (blue; **a**,**c**,**e**; n = 10–14) and 12 weeks (orange; **b**,**d**,**f**; n = 8–11) of HFD. (**g–j**) triglycerides (**g**,**h**) and cholesterol (**i**,**j**) in vAT from the mice were measured after 4 (blue; **g**,**i**; n = 10–15) and 12 weeks (orange; **h**,**j**; n = 10–11) of HFD. Bar graphs are representation of mean ± SEM. **p < 0.01.

In order to investigate whether adipocyte CaSR influenced vAT inflammation in-vivo, gonadal vAT harvested from *AdipoqCre*+ *Casr*^*flox*^* Apoe*^*−/−*^ and *AdipoqCre− Casr*^*flox*^* Apoe*^*−/−*^ mice after 4 and 12 weeks of HFD was lysed and the inflammatory cytokines, cholesterol and triglycerides were measured in the tissue lysates to observe the local inflammatory response and lipid uptake. Surprisingly, there was no change in the levels of inflammatory cytokines chemokine (C–C motif) ligand 2 (CCL2), Interleukin-6 (IL6) and Tumor Necrosis Factor-α (TNF-α) upon adipocyte CaSR deficiency in-vivo (Fig. 3a–f). Furthermore, triglycerides levels in the vAT were not changed (Fig. 3g,h). Interestingly, the amount of cholesterol in the vAT from *Casr*-deficient mice was reduced after 12 weeks of HFD but not after 4 weeks HFD (Fig. 3i,j).

## Discussion

Several studies have demonstrated that activation of CaSR stimulates adipose tissue inflammation in-vitro^24–27^. This study set out to investigate the in-vivo effects of adipocyte specific CaSR on inflammation and atherosclerosis development, by using newly established conditional adipocyte-specific CaSR deficient mice on an hyperlipidemic and atherosclerosis prone background. We unexpectedly could not observe any effects of adipocyte CaSR on vAT inflammation in-vivo nor in early or advanced atherosclerotic lesion development. Besides lesion size, also lesion composition was unaltered as no difference in macrophage or collagen content could be observed. Additionally, lack of adipocyte CaSR did not influence systemic parameters, like total plasma cholesterol and triglycerides or leukocyte numbers. Strikingly, vAT inflammation was likewise not altered upon in-vivo adipocyte specific CaSR deficiency.

Besides maintaining calcium homeostasis, CaSR has been shown to play a role in various other physiological processes. CaSR has been demonstrated to contribute to adipocyte dysfunction in-vitro as Cifuentes et al*.* observed that various inflammatory factors, like IL-6, IL-1β, TNF-α and CCL2, were elevated when CaSR was activated using the calcimimetic Cinacalcet in an adipocyte cell line derived from human liposarcoma (LS14)^26^. This elevation of inflammatory factors also increased the recruitment of monocytes in-vitro, suggesting that CaSR stimulates inflammation. Using a similar approach, it could be shown that besides stimulating inflammation, activation of CaSR in human adipocytes inhibited basal lipolysis in-vitro^26^. Furthermore, CaSR activation elevates pre-adipocyte proliferation^27^ and CaSR activation in human SW872 adipocytes by GdCl_3_ promoted adipocyte differentiation and adipogenesis^24^, again supporting a role of CaSR in adipose tissue formation and accumulation. Although all the above described studies point towards a potential involvement of CaSR as prominent player in adipose tissue formation and inflammation, in-vivo validation was missing so far as all studies were performed in-vitro using adipocyte cell lines. Surprisingly, we could not validate the previously observed in-vitro effects of CaSR in our in-vivo model. Several plausible explanations can be raised to explain this discrepancy. One important difference is the methodology that is used to target the CaSR in the various studies. In our study, we evaluated the effects of long-term ablation of CaSR in mature adipocytes using a genetic approach, while most in-vitro studies have used pharmacological CaSR agonists or antagonists. Not only unwanted and yet unidentified side-effects of this pharmacological targeting can occur, but also the time-frame of CaSR targeting is significantly different. Another important aspect that should be noted is the fact that the target cells are quite different in the various experiments. Not only different adipocyte cell-lines from different origins are used, but several studies are also conducted using pre-adipocytes rather than mature adipocytes, while in our study we only targeted mature adipocytes. The importance of this aspect is stressed by the notion that pre-adipocytes can even become phagocytic under specific stimuli and phenocopy macrophages^28^. Finally, a clear distinction should be made between studies focussing solely on adipocytes and studies like ours focussing on adipose tissue. Adipose tissue does not only consist of adipocytes, but also contains nerve cells, vascular cells, adipocyte progenitor cells, fibroblasts, stem cells and immune cells. Together, these cells release various factors such as inflammatory cytokines or adipokines^5,29,30^, adiponectin^31^, resistin^32^, and many complement components^33,34^. The main cytokines that are produced in adipose tissues are TNF-α and IL6^35^, which have been demonstrated to play an important role in various vascular pathologies like atherosclerosis^36^. A study using a co-culture of adipocytes and macrophages in-vitro demonstrated that there is a vicious loop present between these two cells mediated by TNF-α and free fatty acids that aggravates inflammatory responses^37^. Products released by adipocytes are known to directly target the vessel wall to interfere in the process of atherosclerosis development^8^. This interference is for example caused by endothelial cell activation^38^, smooth muscle cell proliferation^39–41^, changing lipid deposition, immune cell attraction and adhesion molecule expression^42^. The fact that adipose tissue is actually a very complex organ and not solely a collection of adipocytes might be another main reason why the deficiency of CaSR specifically in mature adipocytes in our model did not create a difference in the secretome of the vAT nor in systemic effects.

Further supporting the notion that CaSR targeting in adipose tissue should not be restricted to mature adipocytes in order to induce significant effects is the notion that CaSR also plays an important role in immune cells and vascular cells. For example, recent studies have found that CaSR activates the NLRP3 inflammasome in monocytes, thereby driving inflammation and promoting the development of Rheumatoid Arthritis^43,44^. Additionally, stimulation of CaSR in-vitro in monocyte derived macrophages augments the release of inflammatory cytokines, like IL-1β and TNF-α^45^. Interestingly, one study even found that Ca^2+^ has an additive effect on chemotaxis of monocytes to CCL2, in a CaSR-dependent manner^46^. All in all, these studies clearly demonstrate that CaSR activation promotes inflammation in immune cells. Additionally, CaSR has been associated with increased cell adhesion and migration, which are crucial steps in atherogenesis^47^. This effect was mediated via interaction of CaSR with integrins, which are cell adhesion molecules that are present on endothelial cells and play an important role in the tight adhesion and migration of immune cells into the vessel wall, further implicating CaSR in (chronic) inflammatory processes like atherosclerosis. The fact that our results demonstrate that adipocyte-specific CaSR deficiency does not influence vAT inflammation and atherosclerosis development, raises the suggestion that CaSR expression on other cells in the vAT, like endothelial cells, monocytes/macrophages and adipose progenitor cells, play a more important role in inflammation than CaSR expression on mature adipocytes. Future studies should thereby focus on the exact role of progenitor, vascular or immune cell expressed CaSR to elucidate the effects of CaSR on adipose tissue inflammation and atherosclerosis development.

Another explanation for the lack of effects of adipocyte CaSR deficiency might be the choice of diet as CaSR has also been clearly associated with obesity^48^. In humans it could be shown that obese subjects presented a high sensitivity for calcium, compared with normal-weight controls, which could be speculated to result from greater activity of CaSR^49^. Furthermore, medium conditioned by adipose tissue explants from obese subjects increased CaSR expression in adipocytes and this CaSR expression was positively associated with BMI from the donor^50^. In addition, there is a positive correlation between CaSR mRNA expression in human vAT and fat percentage^51^. Therefore, it is possible that a more extreme condition, like obesity, is needed to distinctively elucidate the role of CaSR on adipose tissue inflammation. These studies will be of major importance as CaSR is currently already being used as therapeutic target in the clinic using calcimimetics like cinacalcet, especially in patients suffering from chronic kidney disease^52^. However, the exact impact of such agents on adipose tissue inflammation and function remains undetermined. Therefore, future studies should also focus on the effects of calcimimetics on adipose tissue inflammation, by treating animals with pharmacological CaSR agonists in different disease conditions like obesity or chronic kidney disease, which could have important clinical implications.

In conclusion, although previous studies demonstrated an important role for CaSR in adipose tissue inflammation in-vitro using pharmacological targeting, the conditional mature adipocyte specific CaSR deficiency in hyperlipidemic mice did not appear to have any effects on vAT inflammation in-vivo nor on early of late atherosclerosis development in *Apoe*^*−/−*^ mice. Future studies are needed to elucidate whether CaSR expression on other adipose tissue related cells or in other disease conditions like obesity play a role in adipose tissue inflammation in-vivo.

## Materials and methods

### Mouse lines and atherosclerosis models

The *Casr-floxed* (*Casr*^*fl/fl*^) mice, kindly provided by Dr. W. Chang (The University of California San Francisco, USA) were backcrossed with C57Bl/6 *Apoe*^*−/−*^ mice. For tamoxifen-inducible, adipocyte cell-specific deletion of *Casr*, *Casr*^*fl/fl*^* Apoe*^*−/−*^ mice were crossed with AdipoqCreER^T2^-expressing mice (“*AdipoqCre*”, *Jackson Laboratory, stock No. 025124)*. To induce *Casr* deletion in mature adipocytes, the mice were injected i.p. with tamoxifen (50 mg/kg body weight, from Sigma-Aldrich and dissolved in Miglyol, Caelo) for 5 consecutive days. Knockout of the *Casr* allele was confirmed using primer-pair combinations detecting the wildtype or floxed allele (5′-GTGACGGAAAACATACTGC-3′ and 5′-CGAGTACAGGCTTTGATGC-3′) or knockout allele (5′-CCTCGAACATGAACAACTTAATTCGG-3′ and 5′-CGAGTACAGGCTTTGATGC-3′) (Supplemental Figure S1a,b) and ddPCR (see details below and Supplemental Figure S1c). A recovery period of 1 week after the first tamoxifen injection was allowed after which mice (mixed gender) were fed a high-fat diet (HFD) containing 21% fat and 0.15–0.2% cholesterol (Altromin 132010, Sniff TD88137), starting at 8–10 weeks of age for 4 or 12 weeks (both genders were used for all experiments). All animal experiments were approved and carried out in accordance with relevant guidelines and regulations (Regierung von Oberbayern, Sachgebiet 54, Germany) and every effort was made to minimize suffering. All experimental protocols were approved and the study was carried out in compliance with the ARRIVE guidelines.

### Analysis of atherosclerotic lesions using histology and immunofluorescent staining

For analysis of mouse atherosclerotic lesions, the aortic root and thoraco-abdominal aorta were used. In brief, hearts with aortic root were embedded in Tissue-Tek O.C.T. compound (Sakura) for cryo-sectioning. Atherosclerotic lesion size was quantified after HE-staining of 5 µm transverse sections and averages were calculated from 3 sections. The aorta was opened longitudinally, mounted on glass slides and en face-stained with Oil-Red-O. Aortic arches with main branch points (brachiocephalic artery, left subclavian artery and left common carotid artery) were fixed with 4% paraformaldehyde and embedded in paraffin. Lesion size was quantified after HE-staining of 3 transverse sections. For analysis of the cellular composition or inflammation of atherosclerotic lesions, sections were stained with an antibody to Mac2 (AbD Serotec). Nuclei were counter-stained by 4',6-diamidino-2-phenylindol (DAPI). After incubation with a secondary FITC-conjugated antibody (Life Technologies), sections were analyzed using a Leica DMLB fluorescence microscope and charge-coupled device (CCD) camera. Collagen was stained using Masson’s Trichrome. Blinded image analysis was performed using Leica Qwin Imaging (Leica Lt.) or Image J software.

### Laboratory parameters and flow cytometry

Leukocyte counts in blood were determined using a Celltac Automated Hematology Analyzer (Nihon Kohden) or counting beads (Invitrogen) during flow cytometry analysis. For flow cytometry analysis, whole blood obtained from the retro-orbital plexus of mice was EDTA-buffered and subjected to red blood cell lysis. Leukocyte subsets were analyzed using the following combination of surface markers: neutrophils (CD45^+^CD11b^+^CD115^−^Gr1^high^), monocytes (CD45^+^CD11b^+^CD115^+^), classical monocytes (Gr1^high^ monocytes), non-classical monocytes (Gr1^low^ monocytes), T-cells (CD45^+^CD3^+^), B-cells (CD45^+^CD19^+^). Cell populations and marker expression were analyzed after appropriate compensation and gating using a FACSCanto-II, FACSDiva software (BD Biosciences) and the FlowJo analysis program (Treestar).

### Tissue lysates

Mouse gonadal visceral adipose tissue was extracted immediately after sacrifice and stored in ice-cold PBS until further processing. Directly after, 25 mg of gonadal visceral adipose tissue was lysed in 500 µl of cell lysis buffer (Cell Signalling Technology, #9803) containing protease inhibitors (Complete Protease Inhibitor Cocktail, Roche) and phosphatase inhibitors (PhosSTOP, Roche) using a Tissuelyser (Qiagen) for tissue disruption and homogenization. Protein concentrations were determined using the DC Protein Assay (BioRad).

### RNA isolation

Total RNA isolation was performed by commercially available RNA isolation kit from Qiagen according to the manufacturer’s protocol. RNeasy miniprep kit was used for RNA isolation from the adipose tissue. The quality (A_260_/A_280_) and the quantitiy (ng/µl) of the RNA was measured by NanoPhotometer N60/N50 (Implen). A ratio of ~ 2 for A_260_/A_280_ was accepted as good quality RNA.

### cDNA synthesis

RNA samples were diluted to the same concentration and the cDNA synthesis was performed via the commercially available iScript cDNA synthesis kit from Bio-Rad according to the manufacturer’s protocol.

### Droplet digital PCR

PCR was performed on QX200 Droplet Digital PCR (ddPCR) system from Bio-Rad. 20 µl reaction mixes were prepared using 10 μl of 2X ddPCR SuperMix for probes (No dUTP) (Bio-Rad), 1 µl of 20× FAM labeled primer/probe for the target gene, 1 µl of 20× VIC labeled primer/probe for the housekeeping gene, RNase-/DNase-free water and cDNA sample. Taqman primers were purchased from Thermofisher Scientific. Droplet generation was performed in the QX200 droplet generator (Bio-Rad) by adding 20 µl reaction mix and 70 µl droplet generation oil for probes (Bio-Rad) onto DG-8 cartridges covered with gaskets (Bio-Rad). 42 µl of the droplet solution (containing up to 20,000 droplets) was transferred to the appropriate PCR plate (Bio-Rad) which was then sealed with a piercable foil using the PCR plate sealer (Bio-Rad). Cycling was performed in the ddPCR cycler with the following conditions: 10 min at 95 °C (enzyme activation), 30 s at 94 °C (denaturation) and 1 min at 60 °C (annealing/extension) for 40 cycles, 10 min at 98 °C (enzyme deactivation). The PCR plate was then proceeded to the droplet reader (Bio-Rad). Analysis was performed on QuantaSoft software (Bio-Rad).

### Plasma and tissue lipid levels

Cholesterol and triglyceride levels were analysed using mouse EDTA-buffered plasma or lysed tissues and quantified using enzymatic assays (c.f.a.s. Cobas, Roche Diagnostics) according to the manufacturer’s protocol.

### ELISA

Inflammatory cytokines (IL6, TNF-α, CCL2) were measured using enzymatic assays (ThermoFisher Scientific) according to the manufacturer’s protocol.

### Statistics

All data are expressed as mean ± SEM. Statistical analysis were performed using GraphPad Prism 8 (GraphPad Software Inc.). After verifying normal distribution via D’Agostino-Pearson omnibus normality test, unpaired Student’s t-test with Welch’s correction or Mann–Whitney test were used, as appropriate. p values < 0.05 were considered as being statistically significant.

## Supplementary Information

Supplementary Information 1.
