# Supplementary material for: Adipocyte calcium sensing receptor is not involved in visceral adipose tissue inflammation or atherosclerosis development in hyperlipidemic Apoe−/− mice
Source: Sci Rep. 2021 May 17;11:10409. doi: 10.1038/s41598-021-89893-y (PMC8128899; doi:10.1038/s41598-021-89893-y)
Supplement: Supplementary file 1 — Supplementary Information 1. [file 41598_2021_89893_MOESM1_ESM.docx]

**Supplemental Information**

**Adipocyte Calcium Sensing Receptor is not involved in visceral adipose tissue inflammation or atherosclerosis development in hyperlipidemic *Apoe^-/-^ mice.***

Sai Sahana Sundararaman^1-3^, Linsey J.F. Peters^1-4^, Yvonne Jansen^4^, Selin Gencer^4^, Yi Yan^4,5^, Sumra Nazir^1-3^, Andrea Bonnin-Marquez^1-3^, Florian Kahles^6^, Michael Lehrke^6^, Erik A.L. Biessen^2, 3^, Joachim Jankowski^2, 3^, Christian Weber^4-5, 7-8^, Yvonne Döring^4-5, 9^, Emiel P.C. van der Vorst^1-5, *^

**Supplemental Figure S1**

**Confirmation of knockout of *Casr* in adipose tissue**

**
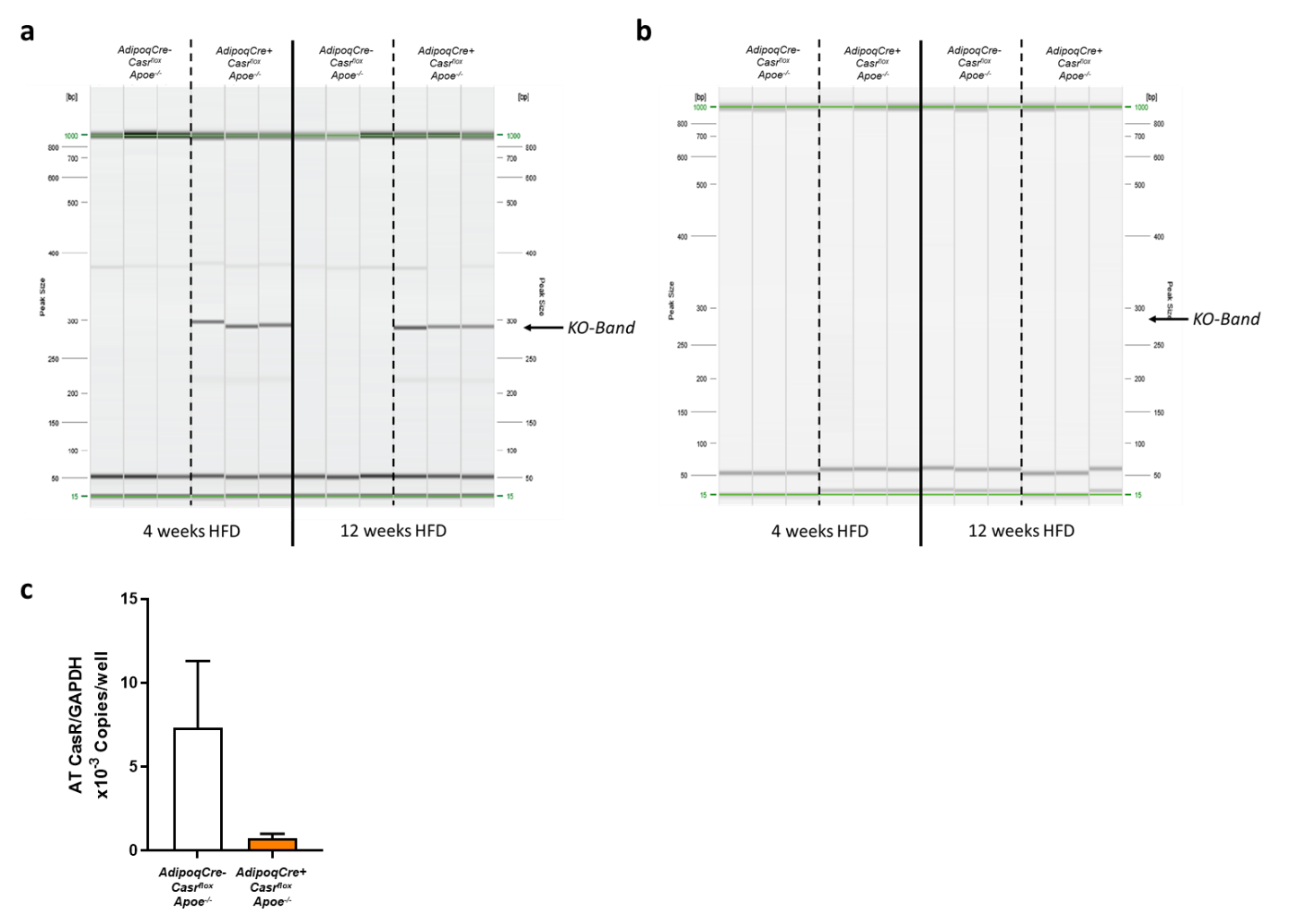
**

**Supplementary figure 1**: **a-b)** Genotyping analysis of the vAT **(a)** and liver **(b)**. vAT isolated from *AdipoqCre+ Casr^flox^ Apoe^-/-^* and *AdipoqCre- Casr^flox^ Apoe^-/-^* mice after 4 and 12 weeks of HFD were genotyped to demonstrate the knockout of CaSR in adipocytes (KO band is present at 280 bp; around 380bp a light non-specific band is observed). **c)** Results from digital droplet PCR analysis, demonstrating Casr expression in vAT from *AdipoqCre+ Casr^flox^ Apoe^-/-^* and *AdipoqCre- Casr^flox^ Apoe^-/-^* mice after 12 weeks of HFD. Analysis has been done using Quantasoft 1.0 (www.bio-rad.com).
